# Sitagliptin, a DPP‐4 Inhibitor, Effectively Promotes the Healing of Diabetic Foot Ulcer: A Randomized Controlled Trial

**DOI:** 10.1111/1753-0407.70156

**Published:** 2025-09-15

**Authors:** Wei Gao, Dawei Chen, Hua He, Nenggang Jiang, Lihong Chen, Xingwu Ran

**Affiliations:** ^1^ Department of Endocrinology and Metabolism West China Hospital, Sichuan University Chengdu China; ^2^ Health Management Center, General Practice Center, West China Hospital Chengdu China; ^3^ Innovation Research Center for Diabetic Foot, Diabetic Foot Care Center, West China Hospital, Sichuan University Chengdu China; ^4^ Department of Endocrinology and Metabolism Hospital of Chengdu Office of People's Government of Tibetan Autonomous Region Chengdu China; ^5^ Department of Laboratory Medicine West China Hospital, Sichuan University Chengdu China; ^6^ Center for High Altitude Medicine, West China Hospital, Sichuan University Chengdu China

**Keywords:** diabetic foot ulcer, dipeptidyl peptidase‐4 (DPP‐4) inhibitors, randomized controlled trial (RCT), sitagliptin, therapy

## Abstract

**Background:**

This randomized controlled trial (RCT) was designed to evaluate the effects of sitagliptin on diabetic foot ulcers (DFUs).

**Methods:**

This was a randomized, open‐label clinical trial. The participants were assigned to either the control group, which received standard conventional therapy alone, or the sitagliptin treatment group, which received an oral administration of sitagliptin (100 mg once daily) in conjunction with standard conventional therapy. The primary endpoints were the ulcer healing rate and adverse reactions. The secondary endpoints included the time to ulcer healing, peripheral blood CD34+ endothelial progenitor cells (EPCs) count, serum levels of stromal cell‐derived factor‐1α (SDF‐1α), and glycosylated hemoglobin A1c (HbA1c).

**Results:**

A total of 62 subjects were enrolled in this trial, with 31 individuals assigned to each group. One participant from each group was lost to follow‐up. Posttrial analysis revealed that, compared with the control group, the sitagliptin group demonstrated a significantly greater reduction in ulcer area and improved efficacy in terms of ulcer healing (*p* < 0.05). Although not statistically significant (*p* = 0.071), the sitagliptin group also tended to have a shorter ulcer healing time. Additionally, the sitagliptin group presented significantly greater numbers of CD34+ EPCs and higher SDF‐1α levels compared to the control group (*p* < 0.05). No statistically significant difference in HbA1c levels was observed between the two groups (*p* > 0.05). No adverse events associated with sitagliptin treatment were reported.

**Conclusions:**

The DPP‐4 inhibitor sitagliptin may facilitate the healing of DFUs independent of its glucose‐lowering effects, potentially by enhancing the mobilization of CD34 + EPCs in peripheral blood.

**Trial Registration:** Registration number: ChiCTR 2000029230, Approval date: 2020/01/19

## Graphical abstract

Sitagliptin promotes diabetic foot ulcer.

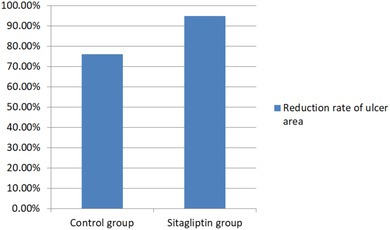

**Summary**

- The randomized controlled trial revealed that the DPP‐4 inhibitor sitagliptin can independently promote the healing of DFUs, irrespective of its glucose‐lowering effects.
- One possible underlying mechanism may involve the mobilization of CD34+ EPCs in the peripheral blood.

## 1 Introduction

Diabetic foot (DF) is a severe complication of diabetes mellitus (DM) and remains the primary cause of nontraumatic lower limb amputations worldwide [1, 2]. DF is characterized by high incidence, amputation, and mortality rates, which not only significantly affect patients' physical and psychological well‐being but also impose substantial economic burdens on families and society. Although the adoption of multidisciplinary care and emerging therapeutic strategies has shown significant efficacy in managing DF [3, 4, 5, 6], there remains a critical need for innovative treatment approaches to enhance wound healing and improve patient outcomes.

Endothelial progenitor cells (EPCs) are a type of adult stem cells that can migrate to ischemic tissues and differentiate into vascular endothelial cells (ECs), thereby facilitating vascular endothelium repair and promoting angiogenesis. Accumulating evidence has consistently shown a significant reduction in EPCs counts among patients with DM, peripheral arterial disease (PAD), and DF [7, 8, 9]. Our previous systematic review and meta‐analysis demonstrated the potential therapeutic efficacy of autologous stem cell transplantation in the treatment of DF [10]. However, given the invasive nature of this procedure, there is an urgent need to develop innovative and clinically feasible strategies capable of effectively mobilizing EPCs into the peripheral circulation and guiding them to ischemic tissues. Thus, targeting modulating EPCs mobilization and function may represent a promising therapeutic approach for managing DF.

Dipeptidyl peptidase‐4 (DPP‐4) inhibitors are extensively prescribed as antihyperglycemic agents in clinical practice. Accumulating evidence suggests that these agents may confer additional therapeutic effects beyond their primary role in glycemic control [11, 12, 13]. Animal experiments and clinical studies have indicated that DPP‐4 inhibitors can increase circulating levels of EPCs [14, 15, 16] and potentially facilitate the healing of diabetic foot ulcers (DFUs) [17, 18]; however, these findings remain preliminary and are subject to debate [19]. To date, no definitive clinical evidence has established a direct correlation between the administration of DPP‐4 inhibitors and improved DFU healing outcomes through enhanced recruitment of EPCs. A proposed mechanism for the potential mobilization of EPCs by DPP‐4 inhibitors may involve the stromal cell‐derived factor‐1α (SDF‐1α)/chemokine receptor 4 (CXCR4) signaling pathway; nevertheless, this hypothesis remains under investigation and lacks comprehensive empirical validation [14, 15, 16].

In this study, we conducted a randomized controlled trial (RCT) to evaluate the efficacy of DPP‐4 inhibitor therapy in facilitating the healing of DFUs and to elucidate the underlying mechanisms involved.

## 2 Method

The study was a randomized, open‐label clinical trial (Registration Number: ChiCTR2000029230, Date: 2020/01/19), conducted with the approval of the Clinical Ethics Committee of West China Hospital, Sichuan University. Written informed consent was acquired from all participants prior to their involvement in this research. The reporting of this trial adheres to the guidelines in the CONSORT statement.

### 2.1 Subjects

Patients aged 18–80 years who were diagnosed with T2DM complicated by foot ulcers located below the ankle joint, classified as Wagner grade 2–4, and with an ankle‐brachial index (ABI) of at least 0.6 were enrolled in this study. All participants had been receiving treatment with either insulin or at least one oral hypoglycemic agent; however, none had received treatment with DDP‐4 inhibitors or GLP‐1 receptor agonists within the preceding 6 months. Individuals with severe comorbidities, including malignancies, significant cardiovascular or cerebrovascular diseases, or severe hepatic and renal dysfunction, were excluded from the study.

### 2.2 Study Design

The study utilized a computer‐generated randomization approach, supplemented by sealed envelopes, to ensure allocation concealment. The experimental design incorporated that blinding was applied exclusively to the personnel responsible for performing area calculations and laboratory evaluator evaluations. The control group received standard conventional therapy, whereas the experimental group was administered an additional daily oral dose of the DPP‐4 inhibitor sitagliptin (Januvia, 100 mg, Merck) in conjunction with standard therapy until the end of the follow‐up period. Conventional therapy consisted of nutritional support, blood glucose control, antihypertensive treatment, lipid‐lowering medications, vasodilators, neurotrophic agents, analgesics, wound debridement, dressing changes, and anti‐infective measures tailored according to drug sensitivity testing or clinical judgment. Glycemic control strategies included patient education, dietary guidance, regular blood glucose monitoring, and the use of hypoglycemic agents. The target HbA1c level for glycemic control was set at 7.0%. Follow‐up was continued until either ulcer healing was achieved or the 12‐week study period concluded, regardless of healing status.

### 2.3 Data Collection and Index Measurement

**FIGURE 1:**
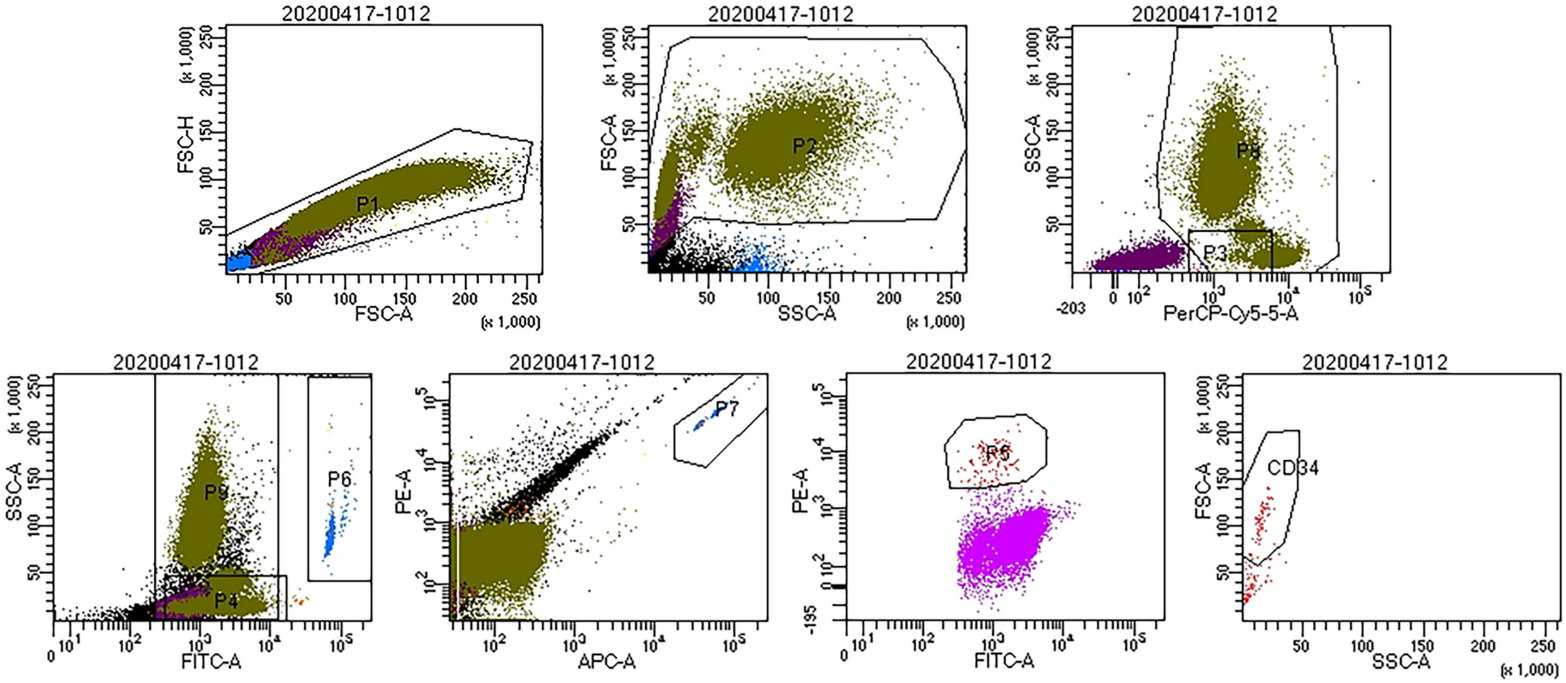
Flow cytometric detection of EPCs in a single subject. (a) and (b) Removal of impurity particles and adherent cells. (c) CD45‐weakly positive mononuclear cells (P3) were selected; P8 represents CD45‐positive leukocytes. (d) Mononuclear cells exhibiting positivity for nucleic acid dye were selected; P9 denotes nucleated cells positive for nucleic acid dye, while P6 indicates microspheres with a fixed value. (e) Fluorescence parameters were introduced, and microspheres were redefined as internal reference targets (P7). (f) CD34+ cells (P5) were identified based on signals meeting both the P3 and P4 criteria. (g) CD34+ cell signals were confirmed as a homogeneous cell component based on FSC/SSC parameters.

Demographic data, personal medical history, family medical history, and current medical history were systematically collected. Fasting venous blood samples were obtained from all participants for comprehensive biochemical analysis. Key parameters, including blood glucose levels, liver and kidney function, serum uric acid concentrations, lipid profiles, and urinary albumin‐to‐creatinine ratio, were measured via an automated biochemical analyzer (ROCHE COBASC702, Shanghai). HbA1c levels were determined via high‐performance liquid chromatography (TOSOH HLC‐723G8, Japan). EPCs were defined as CD34+ EPCs on the basis of established literature [19, 20]. The CD34+ EPCs were quantified via flow cytometry (BD FACSC anto flow cytometry, USA). The gating strategy employed in the flow cytometry analysis is illustrated in Figure 1. Serum SDF‐1α concentrations were determined via a double‐antibody sandwich enzyme‐linked immunosorbent assay (ELISA) (SDF‐1α ELISA kit, EK1818, Signalway Antibody, USA).

### 2.4 Outcomes

The primary outcomes assessed were the ulcer healing rate and the incidence of adverse events. The secondary outcomes included the duration of ulcer healing, the number of CD34+ EPCs in peripheral blood, the serum concentration of SDF‐1α, and the level of glycosylated hemoglobin A1c (HbA1c).

The ulcer healing rate was evaluated on the basis of the percentage reduction in the ulcer area [21]. Complete healing was defined as a 100% reduction in ulcer area. A reduction ranging from 80% to 99% was categorized as significantly effective, a reduction between 40% and 79% was considered effective, and a reduction of less than 40% was classified as ineffective. The percentage reduction in the ulcer area (%) was calculated via the following formula: ([initial ulcer area—unhealed ulcer area] ÷ initial ulcer area) × 100. Only patients who achieved complete healing were included in the analysis of the time required for ulcer healing by the end of the study.

From the time the subjects signed the informed consent form until the completion of the trial, any adverse medical events that occurred were documented as adverse events, regardless of whether a causal relationship with the investigational drug could be established. Adverse events include newly emerging symptoms, signs, diseases, or clinically significant abnormal laboratory test results, as well as preexisting conditions that worsened during the trial. The severity of adverse events was classified into five grades: Grade 1 (mild): asymptomatic or mild symptoms detected only through clinical examination or testing, requiring no intervention; Grade 2 (moderate): symptoms of moderate intensity requiring minimal, noninvasive intervention; Grade 3 (severe): severe or medically significant abnormalities that are not immediately life‐threatening but may require hospitalization, prolong existing hospitalization, or result in persistent or significant disability; Grade 4 (life‐threatening): symptoms or conditions that are immediately life‐threatening and require urgent intervention; Grade 5 (death): adverse events resulting in mortality.

### 2.5 Sample Size

The sample size was estimated via a formula for comparing proportions between two groups, on the basis of our previous data [18]. The formula utilized the following parameters: *k* = 1, *α* = 0.05, *β* = 0.1, and *δ* = *p*_1−_ *p*_2_. The initial results indicated that each group required 27 cases. To account for potential dropouts or missing data, the sample size was increased by 15%, resulting in a final target of 31 participants per group.

$$n_{1}=n_{2}=\frac{{\left(z_{a/2}+z_{\beta }\right)}^{2}\left[p_{1}\left(1-p_{1}\right)/k+p_{2}\left(1-p_{2}\right)\right]}{\delta^{2}}$$

### 2.6 Statistical Analysis

The statistical analyses were conducted using SPSS version 17.0. Continuous variables are presented as means (x̄) or medians (M), depending on the distribution pattern of the data. Data variability was expressed as standard deviation (SD) for normally distributed data and the interquartile range (IQR; P25 ~ P75) for nonnormally distributed data. Comparisons between two independent groups were conducted using either an independent samples *t* test or the Wilcoxon rank‐sum test, depending on the data characteristics. Categorical variables were analyzed via the chi‐square tests. An intention‐to‐treat (ITT) analysis was utilized to handle data from participants who were lost to follow‐up. A two‐sided *p* value less than 0.05 was considered statistically significant.

## 3 Results

A total of 62 subjects were enrolled in this study, with 31 participants randomly assigned to the control group and the remaining 31 to the sitagliptin group. One participant from each group was lost to follow‐up. At baseline, no statistically significant differences were observed between the two groups in terms of sex distribution, age, smoking status, alcohol consumption, history of hypertension, or coronary heart disease, family history of diabetes or hypertension, type of hypoglycemic medication used, aspirin usage, duration of diabetes, systolic and diastolic blood pressure levels, BMI, fasting blood glucose levels, HbA1c levels, blood uric acid levels, lipid profiles, serum creatinine, urinary albumin‐to‐creatinine ratio, or chronic kidney disease (CKD) staging. Additionally, no significant differences were noted in albumin levels or the incidence of complications, including DFUs, between the two groups (all *p* > 0.05; Tables 1, 2, 3). Furthermore, none of the patients underwent percutaneous transluminal angioplasty with stent placement, balloon angioplasty, or surgical interventions.

**TABLE 1:** Comparison of characteristics between the control group and the sitagliptin group.

		Control group	Sitagliptin group	Sum	Statistics	*p*
*n*		31	31	62	—	—
Male		23 (74.2%)	23 (74.2%)	46	0.000	1.000
Female		8 (25.8%)	8 (25.8%)	16		
Age		67.32 ± 10.29	65.06 ± 10.04	66.00 ± 10.17	1.024	0.310
Smoking	Yes	12 (38.7%)	16 (51.6%)	28	1.042	0.307
	No	19 (61.3%)	15 (48.4%)	34		
Drinking	Yes	9 (29.0%)	9 (29.0%)	18	0.000	1.000
	No	22 (71.0%)	22 (71.0%)	44		
Hypertension	Yes	16 (51.6%)	13 (41.9%)	29	0.583	0.445
	No	15 (48.4%)	18 (58.1%)	33		
Coronary heart disease	Yes	5 (16.7%)	6 (20.8%)	11	0.111	0.740
	No	26 (83.3%)	25 (79.2%)	51		
Family history of DM	Yes	12 (38.7%)	11 (35.5%)	23	0.069	0.793
	No	19 (61.3%)	20 (64.5%)	39		
Family history of hypertension	Yes	7 (22.6%)	6 (19.4%)	13	0.097	0.755
	No	24 (77.4%)	25 (80.6%)	49		
Insulin	Yes	31 (100.0%)	31 (100.0%)	62	0.000	1.000
	No	0 (0.0%)	0 (0.0%)	0		
Biguanides	Yes	12 (38.7%)	11 (35.5%)	23	0.069	0.793
	No	19 (61.3%)	20 (64.5%)	39		
Sulfonylureas	Yes	2 (6.5%)	4 (12.9%)	6	1.958	0.162
	No	30 (93.5%)	27 (87.1%)	56		
Alpha glycosidase inhibitors	Yes	4 (12.9%)	4 (12.9%)	8	0.000	1.000
	No	27 (87.1%)	27 (87.1%)	54		
Other hypoglycemic drugs	Yes	2 (6.4%)	1 (3.2%)	3	0.350	0.554
	No	29 (93.6%)	30 (96.8%)	59		
Statins	Yes	28 (90.3%)	29 (93.5%)	57	0.218	0.641
	No	3 (9.7%)	2 (6.4%)	5		
Aspirin	Yes	25 (80.6%)	22 (71.0%)	47	2.026	0.155
	No	6 (19.4%)	9 (29.0%)	15		
Course of DM (month)		15.91 ± 12.32	13.15 ± 9.43	13.99 ± 10.64	1.053	0.321
SBP		139.88 ± 25.32	133.82 ± 23.21	135.42 ± 25.21	0.972	0.235
DBP		78.91 ± 9.31	76.52 ± 12.04	76.93 ± 10.53	0.985	0.329
BMI		24.55 ± 2.42	23.42 ± 1.95	23.85 ± 2.01	1.684	0.246
FPG		9.68 ± 4.96	9.61 ± 4.45	9.64 ± 4.53	0.064	0.932
HbA1c		8.16 ± 1.43	8.40 ± 2.14	8.30 ± 1.83	−0.326	0.665
Blood uric acid		323.98 ± 84.12	305.34 ± 87.34	314.33 ± 85.45	0.812	0.512
TG		1.55 ± 0.45	1.52 ± 0.84	1.53 ± 0.77	0.124	0.895
TC		3.96 ± 1.46	3.69 ± 0.97	3.79 ± 1.20	0.789	0.424
HDL‐C		1.15 ± 0.22	1.08 ± 0.31	1.11 ± 0.32	0.634	0.552
LDL‐C		2.25 ± 1.21	2.03 ± 0.71	2.13 ± 1.03	0.632	0.545
Albumin		40.21 ± 4.18	40.48 ± 4.92	40.33 ± 4.49	0.045	0.951
Serum creatinine		105.32 ± 20.62	111.35 ± 23.32	108.34 ± 22.04	−1.079	0.815
Albumin‐to‐creatinine ratio		212.00 (101.00 ~ 538.00)	219.00 (126.00 ~ 542)	218.00 (118.50 ~ 539.00)	−0.563	0.573
Staging of CKD	CKD1	13 (41.9%)	10 (32.3%)	23	0.622	0.430
	CKD2	13 (41.9%)	16 (51.6%)	29		
	CKD3	5 (16.1%)	5 (16.1%)	10		

**TABLE 2:** Comparison of diabetic complications between the control group and the sitagliptin group.

		Control group	Sitagliptin group	Sum	*χ* ^2^	*p*
*n*		31	31	62	—	—
PAD	Yes	18 (58.1%)	16 (51.6%)	34	0.261	0.610
	No	13 (41.9%)	15 (48.4%)	28		
DPN	Yes	29 (93.5%)	30 (96.8%)	59	0.350	0.554
	No	2 (6.5%)	1 (3.2%)	3		
DR	Yes	19 (61.3%)	17 (54.8%)	36	0.299	0.589
	No	12 (38.7%)	14 (45.2%)	26		
DN	Yes	13 (41.9%)	10 (32.3%)	23	0.622	0.430
	No	18 (58.1%)	21 (67.7%)	39		

Abbreviations: DN, diabetic nephropathy; DPN, diabetic peripheral neuropathy; DR, diabetic retinopathy; PAD, peripheral arterial disease.

**TABLE 3:** Comparison of baseline DFU between the control group and the sitagliptin group.

		Control group	Sitagliptin group	Sum	Statistics	*p*
*n*		31	31	62	—	—
Course of DF(day)		30.00 (20.00 ~ 90.00)	40 (21.00 ~ 80.00)	35.00 (20.00 ~ 70.00)	−0.410	0.682
Sinus tract	Yes	2 (6.5%)	2 (6.5%)	4	0.000	1.000
	No	29 (93.5%)	29 (93.5%)	58		
Wagner 2 grade		4 (12.9%)	3 (9.6%)	7	1.657	0.437
Wagner 3 grade		15 (48.4%)	20 (64.5%)	35		
Wagner 4 grade		12 (38.7%)	8 (25.9%)	20		
Ulcer area (cm^2^)		7.11 (3.19 ~ 11.99)	6.00 (3.12 ~ 11.14)	6.78 (3.14 ~ 11.45)	−0.345	0.730
ABI (left)		0.96 ± 0.34	0.99 ± 0.33	0.97 ± 0.33	−0.432	0.644
ABI(righe)		0.94 ± 0.55	0.96 ± 0.32	0.95 ± 0.41	−0.356	0.784
ABI (mean)		0.95 ± 0.44	0.97 ± 0.33	0.96 ± 0.39	−0.454	0.667
Platelet gel	Yes	18 (66.7%)	19 (70.8%)	37	0.067	0.796
	No	13 (33.3%)	12 (29.2%)	25		

Abbreviation: ABI, ankle–brachial index.

**FIGURE 2:**
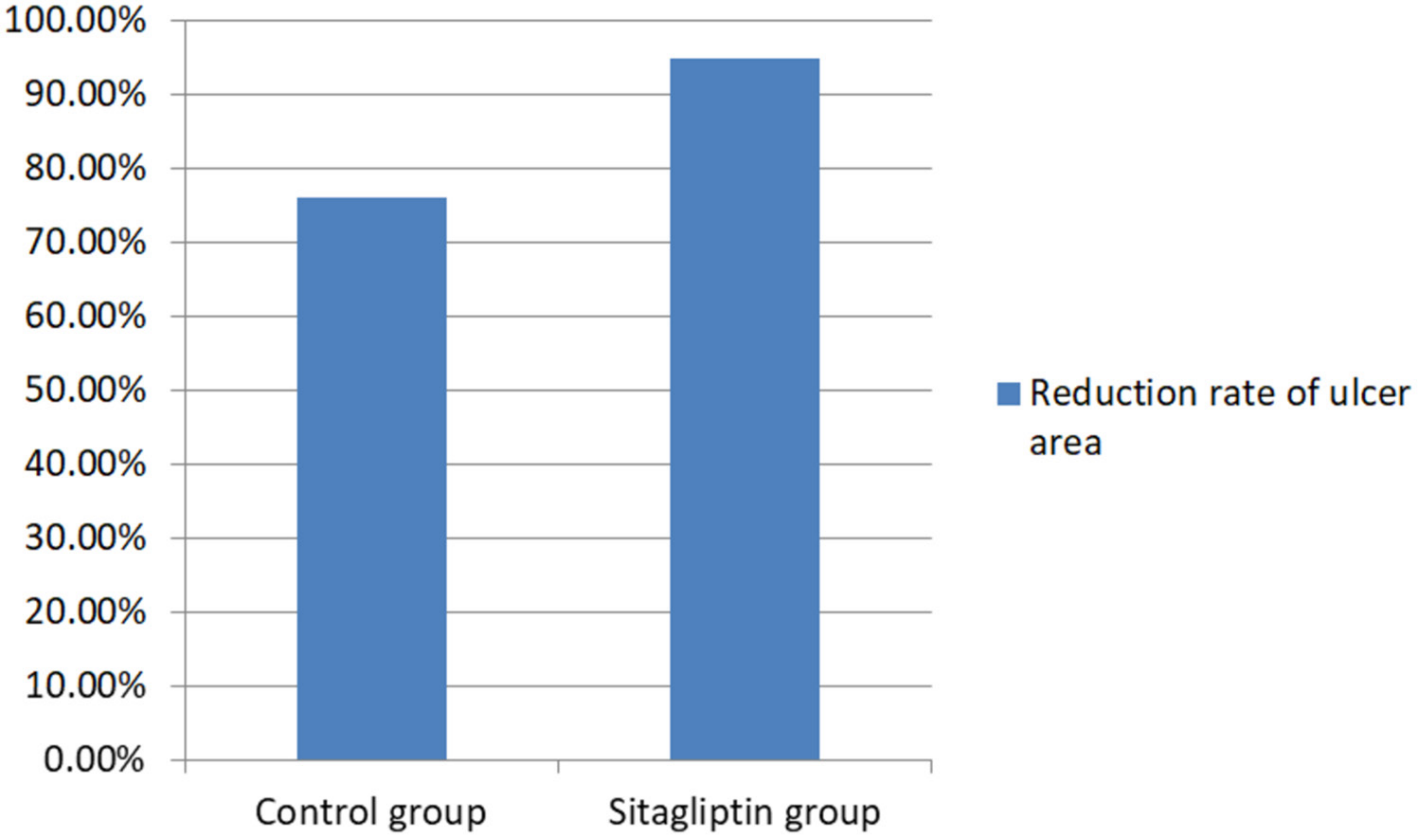
Comparison of ulcer area reduction rates.

Compared with the control group (76.18% ± 33.05%), the sitagliptin group presented a significantly greater mean percentage reduction in the ulcer area (95.00% ± 19.51%) (*p* = 0.019, Table 4, Figure 2). Additionally, the therapeutic efficacy of sitagliptin was superior to that of the control group (*p* = 0.048, Table 4). Although not statistically significant (*p* = 0.071, Table 4), our findings demonstrated that the median time required for ulcer healing in the sitagliptin group (59.00 [47.00 ~ 70.00] days) was relatively shorter than that in the control group (66.00 [54.00 ~ 78.00] days). The progression of ulcer healing in both groups of ulcers is presented in Supporting Informations 1 and 2. No sitagliptin‐related adverse reactions were observed during the course of the study. A summary of all reported adverse events is provided in Table 4.

**TABLE 4:** Comparison of ulcer healing and adverse events between control group and sitagliptin group.

	Control group	Sitagliptin group	Statistics	*p*
*n*	31	31	—	—
Reduction rate of ulcer area(%)	76.18 ± 33.05	95.00 ± 19.51	−2.421	0.019
Efficacy				
Completely healed rate	17 (54.8%)	25 (80.6%)	7.905	0.048
Obvious effective rate	3 (9.7%)	4 (13.0%)		
Effective rate	5 (16.1%)	1 (3.2%)		
Noneffective rate	6 (19.4%)	1 (3.2%)		
Complete healing time(day)	66.00 (54.00 ~ 78.00)	59.00 (47.00 ~ 70.00)	−3.523	0.071
Adverse event	No	No	—	—

**FIGURE 3:**
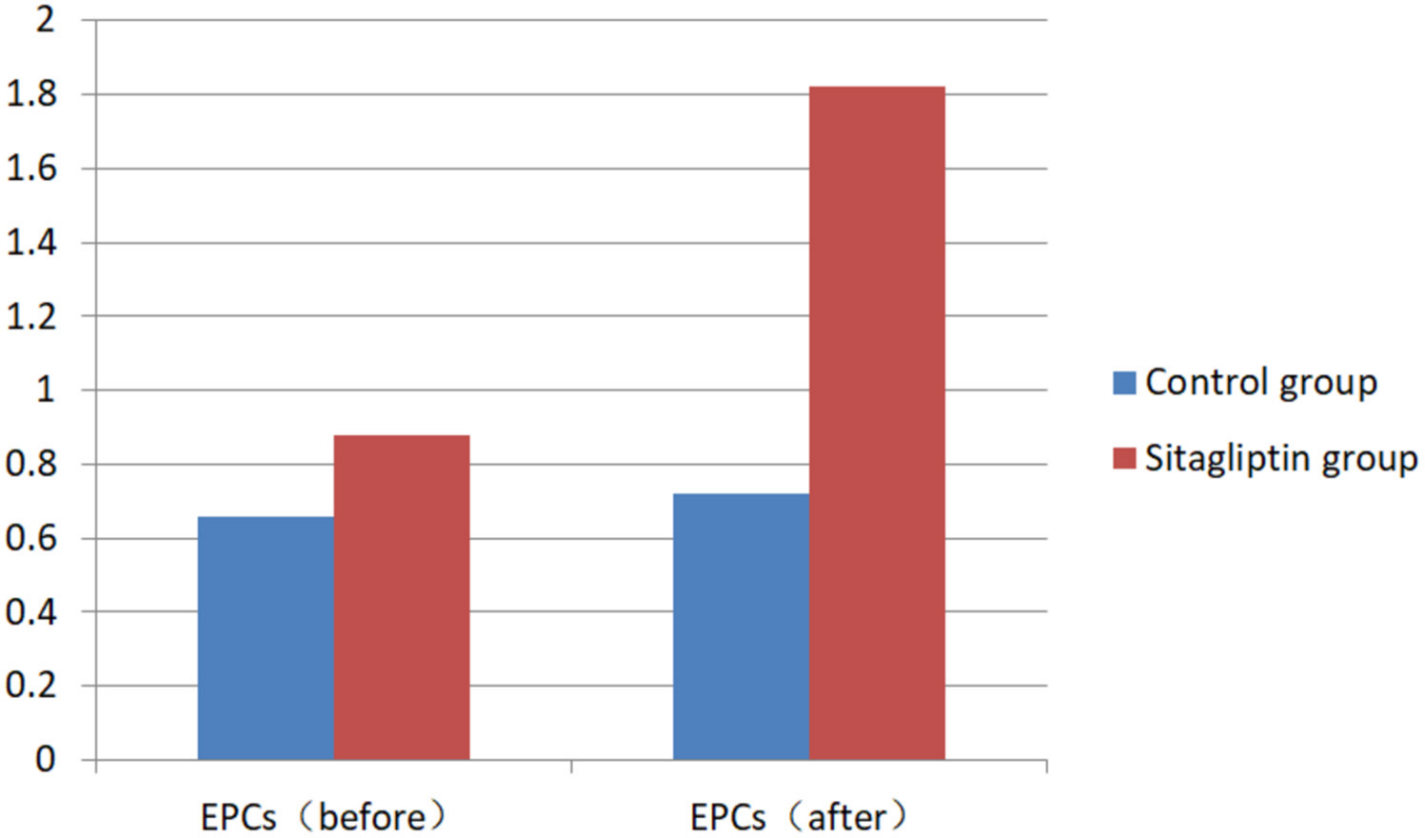
Comparison of the number of EPCs.

There were no significant differences in the baseline levels of CD34+ EPCs in the peripheral blood, serum SDF‐1α concentrations, or HbA1c levels between the two groups. At the end of the trial, no statistically significant difference in HbA1c levels was observed between the sitagliptin group and the control group ([7.04 ± 0.60]% vs. [6.92 ± 0.66]%, *p* = 0.458). Notably, however, the sitagliptin group presented significantly higher levels of CD34+ EPCs (1.82 [1.40 ~ 2.25] vs. 0.88 [0.60 ~ 1.75], *p* = 0.001) (Table 5, Figure 3) and serum SDF‐1α concentrations ([1.60 ± 0.66] vs. [1.06 ± 0.36], *p* < 0.0001) (Table 5) compared with the control group.

**TABLE 5:** Comparison of outcomes in peripheral blood between the sitagliptin group and the control group.

	Control group	Sitagliptin group	*Z*	*p*
*n*	31	31	—	—
EPCs (before)	0.66 (0.36 ~ 0.90)	0.72 (0.40 ~ 0.89)	−0.257	0.572
EPCs (after)	0.88 (0.60 ~ 1.75)	1.82 (1.40 ~ 2.25)	−3.365	0.001
SDF‐1α (ng/mL) (before)	0.98 ± 0.36	0.91 ± 0.23	1.009	0.317
SDF‐1α (ng/mL) (after)	1.06 ± 0.36	1.60 ± 0.66	−3.949	< 0.0001
HbA1c (%) (before)	8.16 ± 1.43	8.40 ± 2.14	−0.326	0.665
HbA1c (%) (after)	6.92 ± 0.66	7.04 ± 0.60	−0.747	0.458

## 4 Discussion

The present study demonstrated that, compared with the control group, the sitagliptin group achieved a significantly greater reduction in the ulcer area and exhibited superior efficacy in promoting ulcer healing. Additionally, the sitagliptin group presented a notable increase in both the number of CD34+ EPCs and the concentration of SDF‐1α compared with those of the control group. Notably, no statistically significant difference in HbA1c levels was observed between the two groups posttreatment.

Sitagliptin is a hypoglycemic agent that has the potential to promote the healing of foot ulcers through its glucose‐lowering effect. However, this study revealed no significant difference in glycated hemolobin levels between the two groups before and after the trial. These findings suggest that sitagliptin may have a beneficial effect on foot ulcer healing through mechanisms independent of its hypoglycemic activity, although the underlying pathways remain to be fully elucidated.

The initial in vivo study [22] conducted in 2012 evaluated the efficacy of a DPP‐4 inhibitor, specifically linagliptin, in promoting the healing of diabetic cutaneous ulcers. To date, only two RCTs [17, 18] have evaluated the effectiveness of DPP‐4 inhibitors for DFUs, both reporting improvements in ulcer healing rates. Our study revealed a complete ulcer healing rate of 54.8% in the control group and 80.6% in the sitagliptin group. These findings are consistent with those of Long's [18] study conducted in China, which did not specify additional ulcer treatment strategies and demonstrated superior outcomes compared with Marfella's [17] study. In our study, some patients received platelet gel therapy to enhance DFU recovery [23]; however, no significant difference in the utilization rates of platelet gel was observed between the two groups. Collectively, the findings of this study, in conjunction with those of previous studies, support that DPP‐4 inhibitors can effectively promote ulcer healing.

The DPP‐4 inhibitors used in previous RCTs [17, 18] were primarily vildagliptin and saxagliptin, which differ from the specific DPP‐4 inhibitors employed in our study. Although three distinct types of DPP‐4 inhibitors have shown therapeutic potential in the treatment of DFUs, the existing body of evidence remains limited, with only a small number of studies reported to date. Therefore, further investigations are warranted to confirm the potential differences in therapeutic efficacy among various DPP‐4 inhibitors for DFUs. Two RCTs have indicated that the potential mechanisms by which DPP‐4 inhibitors may exert their effects on DFUs include the activation of the HIF‐1α/VEGF/iNOS pathway [17] and the induction of EMT [18]. Currently, no clinical trials have been conducted to verify other potential mechanisms by which DPP‐4 inhibitors may exert therapeutic effects on DFUs.

Currently, the prevailing body of research endorses the hypothesis that DPP‐4 inhibitors can augment EPCs recruitment in patients with DM [14, 15, 16, 24, 25]. However, a minority of studies challenge this viewpoint. For example, the combination of saxagliptin and metformin does not increase the quantity of EPCs but instead improves ECs function [19]. Importantly, our study utilized sitagliptin, diverging from previous investigations. To the best of our knowledge, this study is the first to report increased EPC counts in patients with DFUs following treatment with a DPP‐4 inhibitor.

Sitagliptin may enhance the recruitment of EPCs through activating the SDF‐1α/CXCR4 axis, which plays a crucial role in EPCs homing and mobilization. SDF‐1α, also known as CXCL12, functions as a chemokine and interacts specifically with its receptor CXCR4 [26]. EPCs express high levels of CXCR4 [27], while endogenous DPP‐4 cleaves SDF‐1α [28]. By inhibiting the degradation of SDF‐1α, DPP‐4 inhibitors increase its serum concentration. The elevated levels of SDF‐1α subsequently bind to CXCR4 on EPCs, thereby attracting these cells and promoting ulcer healing. Moreover, the SDF‐1α/CXCR4 axis plays an essential role in facilitating the migration of EPCs from peripheral blood to target tissues [29]. Multiple studies have consistently demonstrated that treatment with DPP‐4 inhibitors [14, 15, 30, 31, 32] leads to a significant increase in serum SDF‐1α levels.

EPCs are a type of adult stem cells predominantly located in human bone marrow. In response to tissue ischemia, EPCs are mobilized from the bone marrow into the peripheral blood circulation, where they differentiate into ECs and subsequently colonize ischemic tissues [33]. ECs play crucial roles in the repair of vascular endothelium and the promotion of angiogenesis, as evidenced by extensive animal experiments [20, 33, 34]. Moreover, EPCs can also promote angiogenesis through paracrine mechanisms by secreting growth factors and exosomes [35, 36, 37]. On the basis of these findings, we hypothesize that sitagliptin may facilitate DFU healing by increasing the number of CD34+ EPCs via the SDF‐1α/CXCR4 axis. Additionally, DPP‐4 inhibitors may promote ulcer healing through regulating the MMP/TIMP balance [38, 39] or modulating HMGB1 function [40], suppressing the inflammatory response [41], and activating the NRF2 pathway [42].

In addition to DPP‐4i, several studies have reported the effects of other hypoglycemic agents on DF complications. A meta‐analysis indicated that sodium–glucose cotransporter‐2 inhibitors (SGLT2is) may not significantly improve the prognosis of DF patients [43]. One cohort study [44] revealed that incretin‐based therapies were associated with a reduced risk of DFUs and DFU‐related outcomes, suggesting potential benefits for individuals at high risk of DFUs. Furthermore, metformin has been shown to decrease both the incidence and progression of DFUs [45]. Therefore, these pharmacological options should be considered in patients with DF complications.

Compared with the previous two RCTs [17, 18], this study employed a randomized methodology and implemented blinding procedures for the personnel responsible for performing area calculations and laboratory evaluations, thereby enhancing methodological rigor. However, although no statistically significant difference was observed in Wagner grading between the two randomized groups, the proportion of Wagner grade 4 cases in the control group was greater than that in the sitagliptin group. This discrepancy may influence the study outcomes. The uneven distribution of Wagner grade 4 cases could be attributed to the relatively small overall sample size. Therefore, further investigations should involve more rigorously designed clinical trials with larger sample sizes to confirm these findings. In addition, since all participants in this study were of Chinese ethnicity, the generalizability of the findings to other ethnic populations requires further validation.

## 5 Conclusion

The DPP‐4 inhibitor sitagliptin has been shown to promote the healing of DFUs, independent of its hypoglycemic effects. A potential mechanism underlying this phenomenon may involve the upregulation of CD34+ EPCs in the peripheral blood. However, further research is necessary to substantiate this proposed mechanism.

## Author Contributions

W.G. collected the data, conducted the statistical analysis, and wrote the manuscript. D.C., H.H., and L.C. were responsible for enrolling patients and taking care of patients. N.J. conducted laboratory tests. X.R. was responsible for the study design and manuscript revision.

## Disclosure

The study protocol was approved by the Clinical Ethics Committee of West China Hospital, Sichuan University (2019174).

## Consent

Informed consent was acquired from all participants.

## Conflicts of Interest

The authors declare no conflicts of interest.

## Supporting information


**Supporting Information 1.** One patient in the control group, a 69‐year‐old female, was followed up at A: 0 w, B: 4 w, C: 8 w and D: 12 w.
**Supporting Information 2**. One patient in the sitagliptin group, a 71‐year‐old female, was followed up at A: 0 w, B: 4 w, C: 8 w and D: 12 w.
