# Supplementary material for: Sitagliptin, a DPP‐4 Inhibitor, Effectively Promotes the Healing of Diabetic Foot Ulcer: A Randomized Controlled Trial
Source: J Diabetes. 2025 Sep 15;17(9):e70156. doi: 10.1111/1753-0407.70156 (PMC12434403; doi:10.1111/1753-0407.70156)
Supplement: Supplementary file 1 — Supporting Information 1. One patient in the control group, a 69‐year‐old female, was followed up at A: 0 w, B: 4 w, C: 8 w and D: 12 w. Supporting Information 2. One patient in the sitagliptin group, a 71‐year‐old female, was followed up at A: 0 w, B: 4 w, C: 8 w and D: 12 w. [file JDB-17-e70156-s001.docx]

**
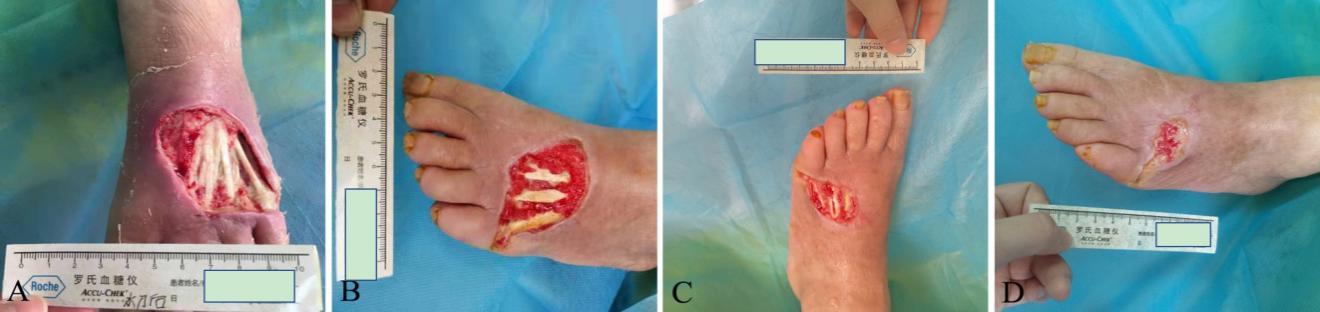
**

**Supplement 1. One patient in control group, female, 69 years old, followed up on A: 0w, B: 4w, C: 8w and D: 12w**

**
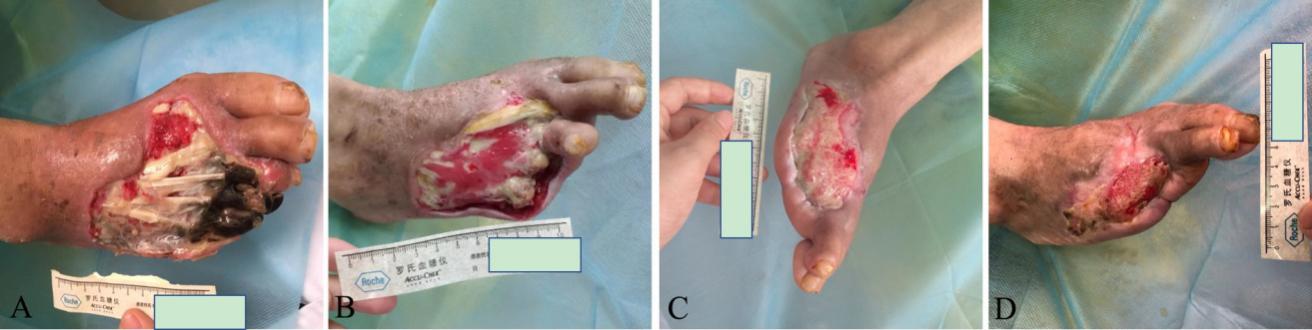
**

**Supplement 2. One patient in sitagliptin group, female, 71 years old, followed up on A: 0w, B: 4w, C: 8w and D: 12w**
